# Improved Passive Gamma Emission Tomography image quality in the central region of spent nuclear fuel

**DOI:** 10.1038/s41598-022-16642-0

**Published:** 2022-07-21

**Authors:** Riina Virta, Tatiana A. Bubba, Mikael Moring, Samuli Siltanen, Tapani Honkamaa, Peter Dendooven

**Affiliations:** 1 Helsinki Institute of Physics, University of Helsinki, Helsinki, Finland; 2 Radiation and Nuclear Safety Authority (STUK), Vantaa, Finland; 3 Department of Mathematical Sciences of the University of Bath, Bath, UK; 4 Department of Mathematics and Statistics of the University of Helsinki, Helsinki, Finland

**Keywords:** Nuclear fuel, Nuclear waste, Applied mathematics, Imaging techniques

## Abstract

Reliable non-destructive methods for verifying spent nuclear fuel are essential to draw credible nuclear safeguards conclusions from spent fuel. In Finland, spent fuel items are verified prior to the soon starting disposal in a geological repository with Passive Gamma Emission Tomography (PGET), a uniquely accurate method capable of rod-level detection of missing active material. The PGET device consists of two highly collimated detector banks, collecting gamma emission data from a 360° rotation around a fuel assembly. 2D cross-sectional activity and attenuation images are simultaneously computed. We present methods for improving reconstructed image quality in the central parts of the fuel. The results are based on data collected from 2017 to 2021 at the Finnish nuclear power plants with 10 fuel assembly types of varying characteristics, for example burnups from 5.7 to 55 GWd/tU and cooling times from 1.9 to 37 years. Data is acquired in different gamma energy windows, capturing the peaks of Cs-137 (at 662 keV) and Eu-154 (at 1274 keV), abundant isotopes in long-cooled spent nuclear fuel. Data from these gamma energy windows at well-chosen angles are used for higher-quality images, resulting in more accurate detection of empty rod positions. The method is shown to detect partial diversion of nuclear material also in the axial direction, demonstrated with a novel measurement series scanning over the edge of partial-length rods.

## Introduction

Finland is constructing a first-of-its-kind underground geological repository for spent nuclear fuel disposal in Olkiluoto, Eurajoki. The start of operations at the new facility is scheduled for 2025^1^. Prior to the disposal, the spent fuel needs to be comprehensively verified to assure that nuclear safeguards objectives are met and that all nuclear material stays in declared use. This was a major driver for the work of designing a Passive Gamma Emission Tomography (PGET) device that was approved by the International Atomic Energy Agency (IAEA) for nuclear fuel inspections. This Non-destructive Assay (NDA) method detects gamma emission from the spent fuel assembly from all angles with two linear arrays of highly collimated CdZnTe gamma detectors^2^. With modern inverse problems techniques, a 2D cross-sectional image is reconstructed^3^. Both the attenuation and the gamma activity of the fuel are simultaneusly computed, and based on the images, even rod-level anomalies in the fuel can be detected. Together with the neutron multiplication verifying method PNAR (Passive Neutron Albedo Reactivity)^4–7^, the fuel material can be verified with very high confidence.

Recent studies on the image reconstruction methods and analysis of PGET data have been conducted worldwide. Fang et al.^8^ have studied iterative reconstruction methods and automated rod identification from simulated fuel data. Shiba et al.^9,10^ have simulated data with Poisson noise and compared different iterative reconstructions methods. Eldaly et al.^11^ have estimated fuel rod activities and their uncertainties with Bayesian approaches. Most authors have limited access to real measurement data, and therefore simulated data is often used. The IAEA has also released a PGET dataset measured with mockup fuel consisting of neutron-irradiated cobalt rods containing Co-60, and this mockup data has been used widely^8,9^.

During 2021 and 2022, PGET measurement data was acquired at the Finnish nuclear power plants. A unique series of measurements was conducted on BWR (Boiling Water Reactor) fuel to test the ability of the PGET method to detect partial diversion also in the axial direction of the fuel. For VVER (Water-Water Energetic Reactor) fuel, new methods were used to enhance the reconstructed image qualities in the central parts of the fuel, both with data acquisition changes as well as with data post-processing tools. Image quality matters were investigated previously in^12,13^ and the results presented here build upon them. The results show that the visibility of empty fuel grid positions in the central parts of the fuel is enhanced with our proposed novel method. The results are compared to measurement data from 2017 to 2020 in general terms to see overarching trends in image quality.

## Materials and methods

### Passive Gamma Emission Tomography device

The Passive Gamma Emission Tomography (PGET) device is an NDA instrument that gathers data from spent nuclear fuel assemblies for tomographic image reconstruction. The PGET device houses two detector banks on opposite sides of a torus. Each detector bank consists of a linear multi-slit tungsten collimator backed by a row of CdZnTe gamma detectors. In this arrangement, only gamma rays travelling in a very specific transaxial direction can be detected, providing transaxial imaging information. A spent fuel assembly is placed in the central hole of the device and tomographic data acquisition is performed by rotating the detector banks a full 360° around it, gathering gamma emission data from all angles. The collimator slits of the device are trapezoidal in shape towards the axial direction, opening up the axial view of a fuel assembly, increasing the gamma ray count rate and allowing for shorter data acquisition times. The axial field of view of the detectors into the fuel is around 20–30 cm, depending on the fuel dimensions. The axial field of view increases from the front to the back of the assembly. The device is described in more detail in^2,14^.

The PGET device has been designed and developed jointly with experts from the IAEA, STUK in Finland and others^15^, and it was approved in 2017 by the IAEA for NDA inspections^16^.

### Measurement data

From 2017 to 2021, the PGET device has been used in collaboration with the IAEA and the European Comission at the Finnish nuclear power plants in altogether seven measurement campaigns. In total 101 individual fuel assemblies have been measured, out of which 12 included fuel grid positions with missing rods. The initial enrichments have ranged from 1.9 to 4.4 %, average burnups (BU) from 5.7 to 55 GWd/tU and cooling times (CT) from 1.9 to 37 a. Campaigns have been conducted at the Loviisa nuclear power plant in 2017, 2018, 2020 and 2021 and at the Olkiluoto nuclear power plant in 2017, 2019 and 2021. The nine Olkiluoto BWR fuel types measured are 9x9-1AB, 8x8-1, ATRIUM 10, SVEA-64, SVEA-100, SVEA-96 OPTIMA, SVEA-96 OPTIMA2, GE-12 and GE-14. At Loviisa, all fuel is of the VVER-440 type. All relevant fuel parameters for the assemblies presented in this work can be found as Supplementary Table S1 online.

The gamma emission data acquired from the measurements is preprocessed first. Misbehaving detectors resulting in abnormalities in the data are removed and an interleaved value from the neighboring detectors is used instead. The profile of the detector counts integrated over the 360° rotation is also smoothed to account for individual differences in the detector responses. After preprocessing, a Filtered Back-projection (FBP) is performed to provide a quick initial reconstruction. Based on this FBP image, the fuel type is deduced and a pre-defined fuel grid based on the declared information of the fuel geometry is fitted onto the FBP image. This will act as a base for the reconstruction of activity and attenuation images, where the grid is used as a prior. Solutions where rod-shaped areas are at the predefined positions are favoured in the reconstruction process, but nothing about the content of these positions is assumed (whether they contain water or fuel, for example). Activity and attenuation images are then reconstructed by solving a constrained regularised problem with an iterative scheme. The mathematical details of the approach used can be found in^3,13^.

Average activity values for each fuel grid position are calculated from the activity images by averaging the pixel values over the area of each rod. These values are then used for classifying the grid positions into empty and filled positions. In this classification, each grid position is compared to its immediate neighbors and a classification border is computed using support vector machines. The support vector machines are trained on the reconstructions of training data sets from varied assemblies including mock-up fuel and real spent fuel assemblies with different rod placements and intensities. The classification process is described in more detail in^3^. The end result of the analysis is a reconstructed 2D image of the fuel and a conclusion about each grid position either being classified empty or filled.

### Image quality index

The quality of the reconstructed gamma activity images is assessed with a tailor-made image quality index, which enables quantitative comparison between different images. The index helps evaluating the effects of different data processing approaches or settings on the end result. It looks into the most important quality of the images: how much the empty fuel grid positions differ from the filled positions.

The image quality index is based on comparing the fuel grid positions where a fuel rod is known to be present to such positions which include only water (or partial-length rods, for the special case of the axial scan). Activity average values of each position are used for the comparison.

**Figure 1:**
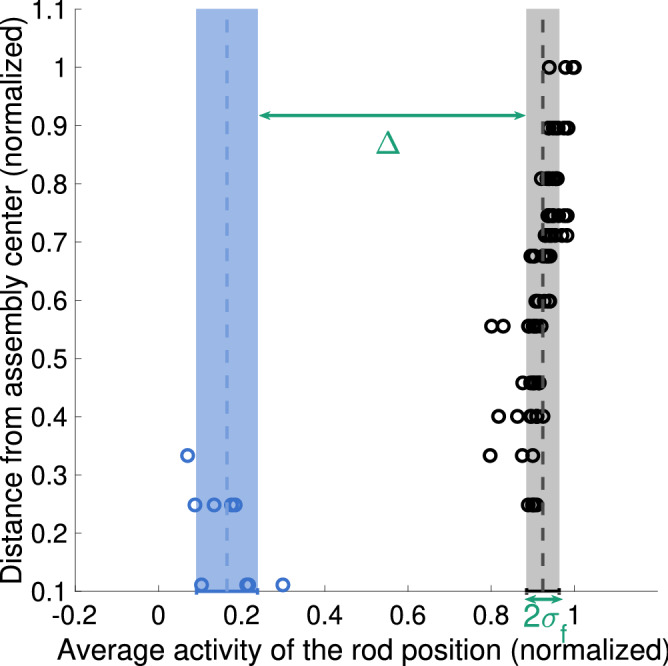
Illustration of image quality index, on a plane of average activity of the fuel grid positions versus the distance from the assembly center. Circles represent individual grid positions, colors denoting the type (black for filled grid position, blue for empty grid position). The mean average activity ($\mu _e$ and $\mu _f$) of both the empty grid positions and the filled grid positions is marked with a dashed line (blue and black, respectively). Standard deviations ($\sigma _e$ and $\sigma _f$) of these two groups are marked with bands of corresponding colors. Twice the standard deviation of the full-length rods is marked with a green arrow and “2$\sigma _f$”, illustrating the second part of the image quality index $[\Delta$, $\sigma _f]$. The “$\Delta$” index is marked with a green arrow.

Figure 1 illustrates how the image quality index is formed. The image quality index is a pair of two values $[\Delta$, $\sigma _f]$. The first of these (“$\Delta$”) is defined as $\Delta \equiv (\mu _f - \sigma _f) - (\mu _e + \sigma _e )$, where $\mu _f$ and $\mu _e$ are the means and $\sigma _f$ and $\sigma _e$ are the standard deviations of the average activity of the filled grid positions and empty grid positions, respectively. The index calculation is restricted to include only full-length fuel rods, because for partial-length rods it cannot be assumed beforehand at which axial position the data has been acquired, and thus, whether the position should be treated as empty or filled.

In an ideal case, the separation between the full-length fuel rods and the empty grid positions in the axial field of view ($\Delta$) is large enough to allow these two groups to be perfectly discriminated. In addition to the activity distance, the standard deviations of both the full-length fuel rods ($\sigma _f$) and the empty grid positions need to be small enough to allow this discrimination. Thus, the larger the $\Delta /\sigma _f$ ratio, the better the separation between empty grid positions and positions filled with active material. The two parts are included in the image quality index to allow a more detailed investigation into the underlying mechanisms of the image quality. Both values are marked in Fig. 1 for clarity. The image quality index can also be used as a purely rod-level measure, if a single fuel grid position is compared to other positions.

Water positions are chosen as the reference point for empty grid positions because most fuel assemblies contain water channels. In our measured data, few assemblies have declared missing rods (only 12 out of 101), but from a methodological point of view, the water positions can also be treated as empty rod positions in the fuel grid and they act as good substitutes for “missing” fuel rods in the method development.

**Figure 2:**
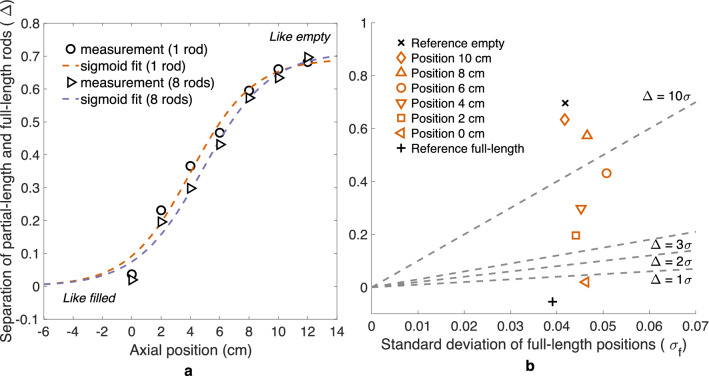
Partial-length rod edge scan results for an ATRIUM 10 assembly (BU 44.3 GWd/tU, CT 13.2 a). (**a**) The difference in average activity between partial- and full-length rods versus axial position of the PGET device, while sweeping across the partial-length rod edge. The axial position is relative to the lowest measurement position. Sigmoid curves are fitted to the data. Data and fits are shown for a case with all 8 partial-length rods included as well as for a case with only one partial-length rod. (**b**) Image quality index plot for the axial sweep over the partial-length rod edge for the same assembly as in (**a**). Black markers denote reference measurements conducted at positions where the partial-length fuel rods are fully visible (“Reference full-length”) and where they are completely out of view (“Reference empty”). Orange markers denote the six different axial positions where scans were made. Acceptance criteria in terms of constant $\Delta /\sigma _f$ are marked with dashed lines.

In image quality plots shown for different measurement results (like Fig. 2b for example), the image quality index is plotted in the $[\Delta$, $\sigma _f]$-plane. The quality of different reconstructed images is compared based on their location in this plane. The image quality index plots show acceptance criteria for the detectability of empty grid positions ranging from 1$\sigma$ to 10$\sigma$, illustrating the amount of separation required between the two groups for accurate classification into empty and filled grid positions. A separation of 3$\sigma$ means that the chance of an empty rod position going unnoticed is less than 1%. If the separation is larger, the two groups can be separated with extremely high confidence.

## Results

In the following we present results from the latest three measurement campaigns, namely from the Loviisa nuclear power plant in 2020 and 2021, and from the Olkiluoto nuclear power plant in 2021.

### Partial-length rod scan

An axial scan over partial-length rod edges was performed on an assembly of type ATRIUM 10 (BU 44.3 GWd/tU, CT 13.2 a) to find out the sensitivity of the PGET method to detect a possible diversion of nuclear material in the axial direction. The geometry of the device (tapering of the collimator slits to allow for reduced data acquisition time) causes the field of view of the device to be rather broad in the axial direction. Thus, changes in the fuel rods in the approximately 20–30 cm axial field of view area will be seen as differences in the activity of the rods in the reconstructed images. If part of a fuel rod is missing and some of that empty space is present in the field of view, the resulting image will show a lower activity of that rod in question.

An axial scan over the partial-length fuel rod edge was performed with seven steps of 2 cm increments. The aim was to estimate how much of the fuel rod needs to be missing before the reconstruction results will differ from the baseline result acquired at a height where the partial-length fuel rods are covering the full field of view of the device.

Figure 2a shows how, for an assembly containing partial-length rods, the difference in average activity between partial- and full-length rods changes with axial position of the PGET device. The separation of partial-length and full-length rods ($\Delta$) versus axial position is plotted for two cases: with all 8 partial-length rods included and with only one partial-length rod included in calculating the values. The latter was made to investigate whether the absence of only one rod would be noticed. The axial position of the edge of the partial-length rods was estimated by fitting a sigmoid curve to the data.

The sigmoid curve used is of the form:

1 $$\begin{aligned} y = \frac{1}{(1+\exp ((b-x)/c))} , \end{aligned}$$

where *x* is the axial position and *b* and *c* are free parameters in the fit, *b* representing the axial position where $y = 1/2$ and *c* indicating the steepness of the slope. The fit is obtained by a least-squares minimizing algorithm.

If the halfway point of the sigmoid curve represents the edge of the partial-length fuel rod, based on the fit the edge would be at $4.85 \pm 1.34$ cm in the relative scale, corresponding to around 2.25 m from the bottom of the assembly. The validity of this estimate method requires further investigation, but the result shows that the PGET device is able to detect a partial diversion of fuel material in the axial direction with a much higher precision than its resolution in that direction (approximately 20–30 cm).

Figure 2b shows the image quality index plot for the partial-length rod edge sweep. The ability to detect axial diversion was assessed by comparing image quality indices for different scans, conducted at different axial positions. A $\Delta /\sigma _f$ ratio of 3 is already obtained for most of the scanning positions, even when only a fraction of the partial-length rods is missing from the field of view of the device.

### Reconstruction image quality enhancement in the central region of the fuel

**Figure 3:**
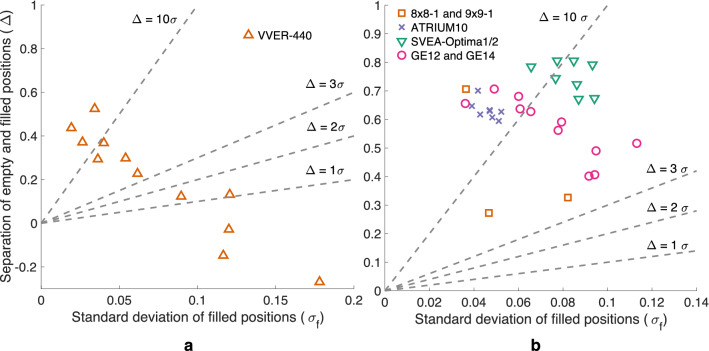
Image quality index plots for images from Loviisa 2020 (**a**) and Olkiluoto 2021 (**b**) measurement campaign results. Different fuel types are denoted with varying colors and markers. Note that the Loviisa 2020 results are from a gamma energy window of 600–700 keV and the Olkiluoto 2021 results from a 650–700 keV window.

Figure 3 illustrates how the ability of the PGET method to detect missing fuel rods in the central parts of the fuel has not yet been fully demonstrated for the VVER-440 fuel, although the method performs well on the BWR fuel. For BWR fuel (Fig. 3b), half of the assemblies satisfy the criterion of 10$\sigma$ and all assemblies satisfy the 3$\sigma$ criterion. For VVER-440 fuel (Fig. 3a) it is clear that the quality of the reconstructed images is not good enough: only one fourth of the assemblies satisfies the 10$\sigma$ criterion and one fourth even fails the 1$\sigma$ criterion. The three worst-behaving assemblies have a negative image quality index value $\Delta$, meaning that the water channel positions show a higher activity than their surrounding rods.

The location of water channel positions might have a minor influence on the results. For the BWR assemblies, there is a varying number of water channel positions in the grid and in the VVER-440 fuel there is only the one position in the center. For the image quality index, these positions are treated equally and if the assembly has multiple water positions, a mean value is used. For BWR assemblies, which often have water channel positions a bit further out from the very center, the visibility of these positions for the gamma ray detectors is better. The VVER-440 assemblies thus have a disadvantage due to their geometry, when they are compared with the image quality index of BWR assemblies.

Based on this comparison it is clear that the quality of the images especially in the central region needs to be enhanced. In the remainder of this section we describe data acquisition changes implemented to better capture the desired gamma peaks and post-processing was fine-tuned for this specific application.

#### Narrower energy windows

In the past, data acquisition has usually been done in a standard energy window of 600–700 keV, capturing the 662 keV peak of Cs-137, the most intense gamma ray emitted by long-cooled fuel. This relatively wide window also captures a lot of photons scattered to lower energies, and while these photons contribute to the overall statistics, the information that they carry about the spatial position of the fuel rods is low. Thus, the energy window was cut narrower to test if the quality of the data could be improved by only including gamma rays in the photopeak.

The energy resolution of the CdZnTe-semiconductor detectors used in the PGET device is 10.7 ± 0.4 keV FWHM (full width at half maximum) at 662 keV. This allows for a tighter gamma energy window around the 662 keV full-energy peak than the previously used 600–700 keV, but the resolution should be kept in mind when fine-tuning the window. The narrowest window which captures a very large fraction of the full-energy events is about 10 keV around 662 keV. We therefore chose a 650 keV lower limit for the window, eliminating as many scattered gamma rays as possible. The upper energy limit is not as critical, because there are much less counts at energies right above the 662 keV peak than right below it. We chose an upper limit of 700 keV in order to be compatible with the previously chosen upper limit for the Cs-137 energy window.

**Figure 4:**
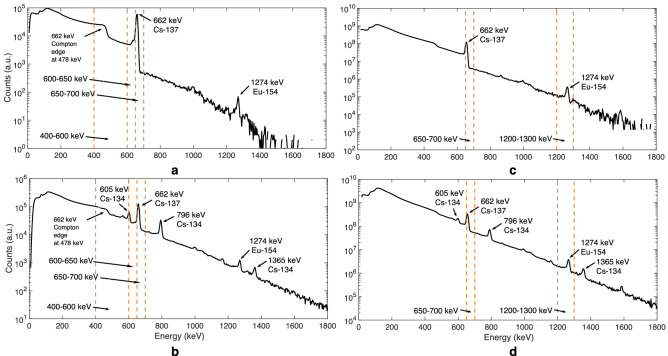
Gamma energy spectra and energy windows used (marked with orange dashed lines) for two BWR assemblies (left) and two VVER assemblies (right). (**a**) $8 \times 8-1$ (BU 18.6 GWd/tU, CT 37 a). (**b**) SVEA-96 OPTIMA2 (BU 43.6 GWd/tU, CT 5.2). (**c**) VVER-440 (BU 34 GWd/tU, CT 29 a). (**d**) VVER-440 (BU 44 GWd/tU, CT 7.2 a). The isotopes responsible for the most intense gamma peaks are indicated.

The gamma energy spectra for two BWR assemblies are shown on the left in Fig. 4. The energy windows used are marked with dashed orange lines and the most intense gamma peaks are pointed out. The commonly used 600–700 keV window is split into 600–650 keV and 650–700 keV windows. The Cs-137 peak is fully captured in the 650–700 keV window and the 600–650 keV window contains Compton scattered photons.

**Figure 5:**
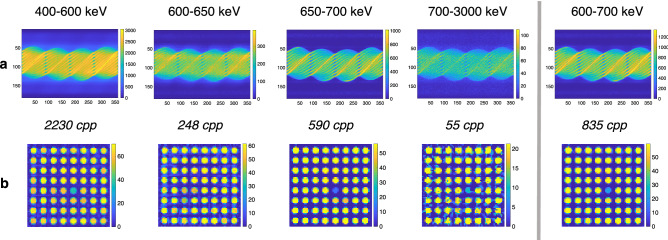
Sinograms (**a**) and reconstructed gamma activity images (**b**) from different gamma energy windows for the same $8 \times 8-1$ assembly as in Fig. 4a (BU 18.6 GWd/tU, CT 37 a). Average counts per sinogram pixel in each energy window are listed underneath the sinograms.

Figure 5 illustrates the effect of the gamma energy window narrowing, when the sinograms and reconstructed images from different energy windows are shown for the same $8 \times 8-1$ assembly as shown in Fig. 4a. The average counts per sinogram pixel in each window are listed underneath each sinogram. The combined window 600-700 keV is shown on the right; this is the window that was previously used for reconstructions.

The 650–700 keV window captures the 662 keV photopeak, representing unscattered Cs-137 photons and providing the sharpest image due to the excellent imaging information that the photons carry. The highest window 700–3000 keV contains the Eu-154 photopeak, which also represents unscattered photons, but with short data acquisition times as in this measurement, the Eu-154 does not produce enough counts for a high-quality image.

The 600–650 keV window mostly captures 662 keV photons from Cs-137 which are scattered inside the fuel grid before entering the detector and thus carry very poor image information and do not contribute to the overall quality of the data in a positive way. The water channel is not visible in the image reconstructed from these counts. Leaving out this part of the data brings the overall counts down, the counts in this window being a third of the counts in the 600–700 keV window. The poor imaging information of these counts, however, causes the quality of 650–700 keV image to be even better than that of the image from the combined 600–700 keV window.

The 400–600 keV window contains only scattered photons. The Compton edge at 478 keV, representing the escape from a detector of the backscattered photon, is most clearly seen in Fig. 4, left. This Compton edge represents the highest energy a Compton scattered electron from a 662 keV gamma ray can achieve. Noteworthy is also that the water channel position is quite clearly visible in the 400–600 keV window, although it is not visible at all in the 600–650 keV window. This is most likely due to the Compton edge that is captured in the 400–600 keV window. As the photons contributing to this edge have not undergone scattering before reaching the detector, they carry good directional, and thus imaging information. Thus, a decent image could also be obtained by collecting data in a narrower energy window capturing the Compton edge only.

**Figure 6:**
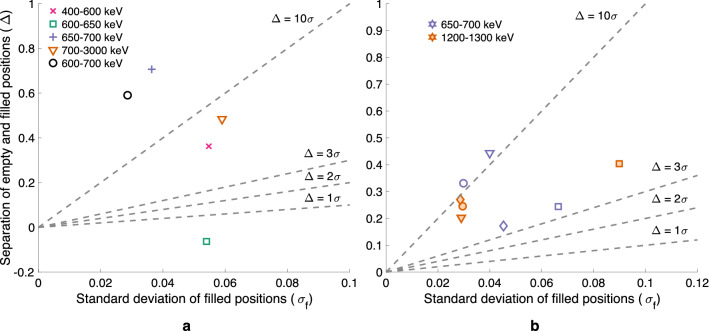
Image quality index plots for images from the Olkiluoto 2021 and Loviisa 2020 measurement campaign results. (**a**) Same Olkiluoto 2021 $8 \times 8-1$ assembly as in Fig. 5 (BU 18.6 GWd/tU, CT 37 a). Results shown in five different gamma energy windows, denoted with varying colors and markers. (**b**) Four different VVER-440 assemblies from Loviisa 2021 campaign, both in the 650–700 keV window (orange) as well as the 1200–1300 keV window (violet). Each assembly has a unique symbol.

Figure 6a shows an image quality index plot for the different energy windows for the same assembly discussed earlier. The optimal energy window 650–700 keV shows approximately the same $\Delta /\sigma _f$ ratio as the usual 600–700 keV window, but the individual values of $\Delta$ and $\sigma _f$ show differences. The $\sigma _f$ is smaller for the 600–700 keV window, probably due to better statistics. However, the $\Delta$ is larger for the 650–700 keV window, indicating a better separation between the empty and filled grid positions in this window.

The quality of images in the 400–600 keV and 700–3000 keV windows is surprisingly good, owing in the first case to the Compton edge and in the latter case to the high-energy full-energy peak from Eu-154. For the 600–650 keV window, the quality is very poor and the water channel position is actually showing as a higher-activity spot compared to the filled positions.

#### Europium window

In addition to the very abundant Cs-137 isotope, also Eu-154 has a significant intensity peak in long-cooled spent nuclear fuel. With a half-life of 8.6 a, this isotope is often seen next to the most abundant Cs-137 with a half-life of 30 a. As the energy of the Eu-154 gamma ray is 1274 keV, it has the potential of revealing the inner parts of the fuel assembly better than the lower energy 662 keV of Cs-137, since it has a bigger chance of not being absorbed in the fuel matrix and reaching the detector. The total attenuation coefficient in UO${_2}$ for 1274 keV is only half of that for 662 keV.

This new approach for central part image quality enhancement was investigated during the Loviisa 2021 measurement campaign. The fuel was measured at a gamma energy window of 1200–1300 keV to capture the 1274 keV gamma peak of Eu-154. Data were also acquired at the 650–700 keV window for comparison. Data acquisition times needed to be lengthened significantly to account for the lower abundance of the Eu-154 isotope in the fuel.

The gamma energy spectra for two VVER-440 fuel assemblies are shown in Fig. 4 on the right. The energy windows used are marked with dashed orange lines, showing both the 650–700 keV as well as the 1200–1300 keV windows. The desired photopeaks are captured in these windows, although the windows could be narrowed further.

**Figure 7:**
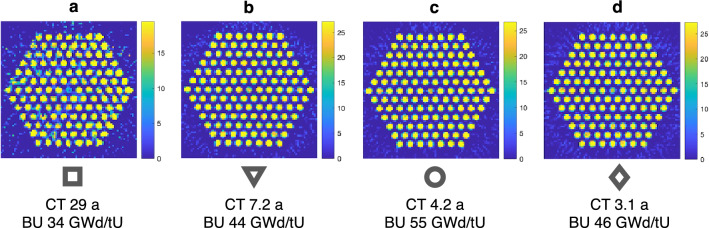
Reconstructed gamma activity images in the 1200–1300 keV window for the same four VVER-440 assemblies (**a**–**d**) as in Fig. 6b. Symbols used in Fig. 6b to denote the assemblies are shown below each image here, along with the CT and BU information.

Figure 7 shows reconstructed gamma activity images in the 1200–1300 keV window for four VVER-440 assemblies with varying cooling times and burnups. The images show that the visibility of the central water channel is poor and that with a long cooling time, as in Fig. 7a, the image is grainy.

Figure 6b shows image quality indices for the four VVER-440 assemblies in both the 650–700 keV as well as the 1200–1300 keV window. The plot shows that the Eu-154 window does not necessarily improve the results compared to the Cs-137 window, at least not with this low number of counts. However, the results show promise for improvements if longer data acquisition times would be used, because the images produced with these low count numbers are unexpectedly good.

The average counts per sinogram pixel in the 650–700 keV window with the long data acquisition time is between 24000 and 32000 for the younger fuel assemblies with cooling times from 3 to 7 a and burnups from 44 to 55 GWd/tU. For the oldest and lowest-burnup assembly (CT 29 a, BU 34 GWd/tU) the average counts per sinogram pixel is around 7600. In the 1200–1300 keV window, these averages are from 420 to 1800 counts per sinogram pixel for the younger fuel and 30 counts per sinogram pixel for the old fuel. For comparison, in the 650–700 keV window with usual data acquisition times the averages would be from 2900 to 3800 counts per sinogram pixel for the younger fuel and around 900 for the old fuel.

The burnup and cooling time of the assemblies will have an effect on the quality of the images reconstructed from these two energy windows. For the outlier in Fig. 6b (square) the assembly is of a lower burnup and longer cooling time (29 a) compared to the others, which have short cooling times (3–7 a) and higher burnups. This lower burnup and longer cooling time assembly has intrinsically lower activity, thus causing less counts leading to poorer statistics. In addition, the large difference in the Eu-154 and Cs-137 half-lives causes the europium intensity to decrease much more rapidly compared to that of the cesium. Also possible inhomogeneities caused by varying conditions in the reactor during operation have had less time to even out, because the burnups are lower. The inhomogeneity results in a larger sigma for the fuel rods.

Image quality indices of reconstructed images show that the quality of the Eu-154 window images is, for most assemblies, at roughly the same level as for the Cs-137 window, although the counts in the higher energy window are significantly lower than in the lower window. The data acquisition times could be further lengthened to get a better idea of whether the Eu-154 window really does bring more information from the center of the assembly. The matter requires further investigation with even longer data acquisition times. It is to be noted that the time required can be very long if similar counts per sinogram pixel are wanted as for the Cs-137 window. For example, for the bottom right assembly (d) in Fig. 4, the data acquisition time would be more than ten times that of a normal measurement.

#### See-through directions

The reconstructed images are usually computed by using 120 equidistant projections from the total of 360 angles. Using all angles has sometimes shown worse results than using fewer angles.

**Figure 8:**
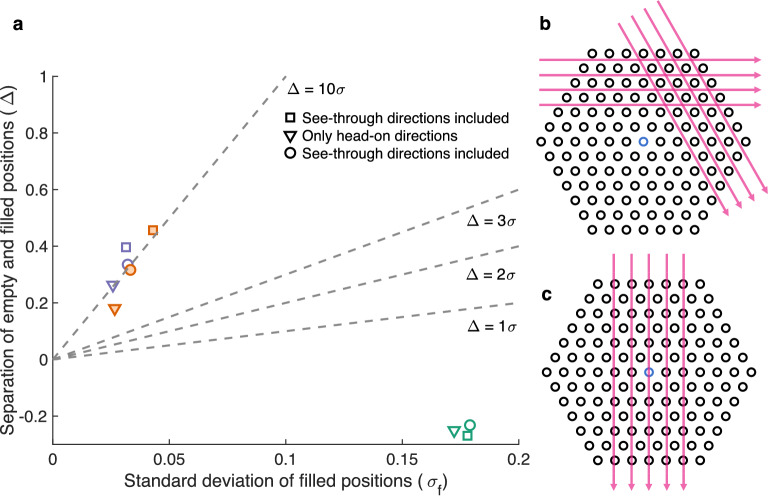
(**a**) Image quality index plot for images of VVER-440 assemblies from Loviisa 2020 with reconstruction directions that include or do not include the see-through directions. Images with see-through directions included are marked with squares and circles. Images where only head-on directions are used in the set are marked with triangles. The three colors denote three different assemblies. (**b**) The so-called “see-through directions” for a VVER-440 assembly. Two of the six see-through directions from which the detectors see right between the rod rows are marked with arrows. (**c**) A “head-on direction”, where the line of sight is always blocked by fuel rods is marked with arrows.

**Figure 9:**
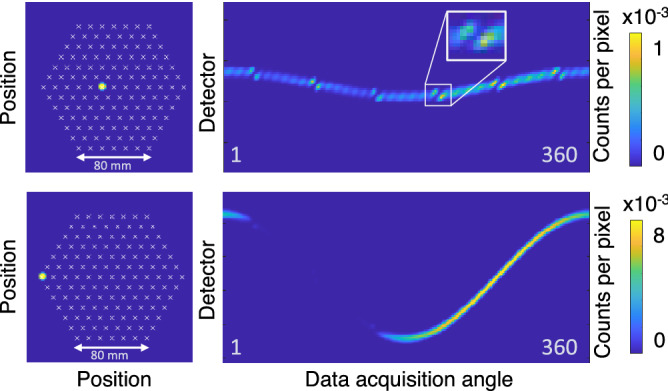
Activity images for two single emitting rod sinogram calculations are shown on the left, an emitting rod near the center (top row) and an emitting rod at the outer edge of the assembly (bottom row). White crosses mark the fuel rod centers for the rods that only attenuate. These activity images are a basis for the sinograms shown on the right, constructed with the forward model used in the reconstructions. Note the scale of the counts values (only 8 times more for the rod at the outer edge). The inset shows a zoom-in around the position of see-through directions.

There are three ways to choose the 120 equidistant angles, and only some of these choices include the so-called see-through directions. These directions are such angles that the detector banks are perpendicular to a direction where there are no fuel rods back to back in some lines of sight, as illustrated in Fig. 8b,c. Angles close to the see-through directions provide a direct line of sight to the central part of the fuel assembly, which at other angles does not contribute to the gamma emission reaching the detectors. This is further illustrated in Fig. 9.

The image quality plot in Fig. 8a shows the effect of see-through directions in the reconstructed images of VVER-440 fuel. Data from three assemblies was reconstructed with all three 120 projection sets available. The first set (square) and the third set (circle) include all the see-through directions but the second set (triangle) does not include any of them, only so-called “head-on directions”. The results with only head-on directions are in two of the three cases (orange and violet) worse compared to the results with see-through directions included in the projection set. In the third set (green) the quality of the image is poor to begin with and then the chosen angles do not have a major effect.

The effect of burnup on the image quality is evident in Fig. 8, just as it was in Fig. 6. The results for the assembly marked with green are notably poor compared to the results for the other two assemblies. The burnup for the green assembly is 13 GWd/tU, whereas for the orange and the violet assembly it is 46 Gwd/tU and 55 Gwd/tU, respectively.

See-through directions were studied further with the help of forward model calculations to understand their meaning for reconstructed image quality. The forward model used in the reconstructions was utilized for calculating different mock-up cases. For a test assembly with all fuel grid positions filled with fuel rods, but only one position housing an emitting spent nuclear fuel rod (see Fig. 9), the effects of the attenuation inside the fuel grid are obvious. As can be seen in the bottom row, from certain angles the signal from the emitting fuel rod is completely blocked by the assembly.

The zoom-in inset in Fig. 9 shows how the gamma signal is momentarily blocked by the rows of other fuel rods, when the device spins around the assembly and is viewing the assembly near a see-through direction. What is noteworthy is that just before and after the blocking from the fuel rod row, the signal is maximal due to the direct line of sight to the center of the fuel. The detectors are in those positions viewing a large amount of other “cheeks” of the fuel rods in the row, which all contribute to the gamma signal measured. Thus, the projections near these angles carry a major part of the information from the central parts of the fuel and are crucial for a good reconstructed image.

## Discussion

The results from the scan over the edge of partial-length fuel rods demonstrate that the PGET method is able to detect partial-length fuel material diversion also in the axial direction. For safeguards conclusions, especially in the Finnish geological disposal context, this is a useful ability, and it could be of use in other studies as well, for example in diagnostics of damaged fuel rods. The structure of fractured fuel rods could be investigated with the method in a non-destructive manner to find out details about the status of the fuel. With the current arrangement of collimators, the PGET device is not the most optimal to this use case, but it could be used in absence of more targeted instruments. With a slight modification of the collimators, the device could be tailored to this kind of use in the future. The highly tapered collimator slits could be narrowed to provide a tighter axial focus, but possibly requiring an increased data acquisition time.

Investigations with the narrower energy windows capturing the Cs-137 gamma peak at 662 keV proved the usefulness of the photopeak. Despite the lower number of counts in the 650–700 keV window compared to the 600–700 keV window, the quality of the reconstructed images is similar or improved, especially in terms of detecting possible empty positions in the inner parts of the fuel grid. The energy window used could still be fine-tuned to be tighter around the desired 662 keV peak. However, one needs to bear in mind the accuracy and stability of the energy calibration and choose the energy window so that a small shift in the calibration would not result in a complete loss of the photopeak. Such shifts are known to sometimes occur in semiconductor detectors due to changes in temperature or count rate. The energy window used in the data acquisition is set for the whole device at once, and since there is a large number of detectors, the energy window has to be wide enough to take into consideration the energy resolution of the worst individual detector.

Results from the higher-energy gamma window containing the Eu-154 show promise in possible image quality enhancements in the central parts of the fuel, because the higher energy of the Eu-154 gamma ray allows it to penetrate through the fuel matrix more efficiently. The linear attenuation coefficient for the Cs-137 gamma ray (662 keV) in uranium dioxide is around 0.16 mm$^{-1}$, whereas for the Eu-154 gamma ray (1274 keV) it is around 0.07 mm$^{-1}$. This means that for a 662 keV gamma ray travelling through 3 fuel rods inside the fuel grid (best case scenario for gamma rays originating from the center of a VVER-440 fuel assembly), only 2.5% make it to the detector when only attenuation is taken into account. For 1274 keV, around 20% of the gamma rays make it to the detector. The difference is notable and sheds light on the fact that the reconstructed images from the Eu-154 energy window are very good even with a fraction of the counts that are usually needed for a good quality image using the Cs-137 gamma rays. The issue with the Eu-154 gamma energy window (1200–1300 keV) is the lower number of counts detected, which causes an unavoidable data acquisition time increase. However, this energy window could be used in the context of geological disposal safeguards if the results from the usual Cs-137 energy window remain inconclusive.

Including the see-through directions in the set of projections used for the image reconstruction has shown promise to increase the image quality in the central parts of the fuel. Peeking from in-between the fuel rod rows is the only way to get a signal directly from the innermost parts of the fuel grid. When investigating the see-through directions for a specific arrangement of fuel rods in a grid imaged with the PGET device, the opening angle of the collimator slits needs to be taken into account. The collimator slits are 1.5 mm wide and 100 mm deep and the detectors are located behind them. Theoretically, the opening angle that each detector sees is 0.86°, and thus a bit smaller than the 1° effective rotation step. This affects which part of the fuel is seen at each direction. In real life, the manufacturing of the collimator slits (alignment and thickness) is never optimal and the collimators are not perfect. However, the scheme for choosing the projections might need to be further investigated and the equidistant angles approach might need to be reconsidered. In the future, measurements will be conducted with a finer angular resolution of 0.5° to investigate the effect.

The sinogram calculations with one emitting fuel rod illustrate the fuel grid geometry effects on the data that is gathered. The minor but significant contribution to the sinogram that comes from the central parts of the fuel assembly shows the characteristics of the grid and the fundamentally important blocking by the fuel rods themselves. As the detectors rotate around the assembly, the view to the center is blocked by other fuel rods every 60°, but before and after these full blocks, the gamma signal is significantly enhanced. The maxima appear on both sides of each of the full signal blockages, but only very narrowly. Missing the optimal spot for the high signal within the selected angles will degrade the image quality significantly, and thus the projections for reconstruction need to be chosen with care. It is also noteworthy that in the sinogram constructed when the lone emitting rod is in the inner part of the fuel, no pixel is as bright as some pixels are in the sinogram where the emitting rod is at the outer edge of the assembly. This further emphasizes the need to capture the essential parts of the data from the inner region, because the rods at the outer edge have an even higher weight on the end result.

The image quality index is a novel measure introduced for this work, mainly to allow comparison between reconstructed images of spent nuclear fuel. The focus of the index is to indicate how well the empty fuel grid positions are distinguished from the positions with fuel. The spent nuclear fuel rods have natural variations in their burnups that originate from varying conditions during operation in the reactor core. Intrinsic deviations in burnup are thus natural, and the activities of individual rods in an assembly are never perfectly homogeneous. The ground truth of the fuel activity is not known and thus there is no way of telling how good the reconstructed image is in absolute terms. In practice, separating the two groups of grid positions (“empty” and “filled”) is tricky due to intrinsically less active rods in the fuel or fluctuations in the images. To exclude effects from possible burnable absorber rods, the location of these rods should be known. Otherwise burnable absorber in the rods might bring the activity values unnecessarily down and interfere with the comparison. For the future, grid positions with burnable absorber material could be left out from the image quality computation.

The effect of the image quality enhancements on the misclassification frequency is noteworthy. For Olkiluoto 2021 data, a full $100\%$ of empty grid positions were correctly classified and $99.2\%$ of filled rod positions were correctly classified. For the Loviisa 2020 and Loviisa 2021 results combined, the empty grid position correct classification rate was $88.0\%$ and the filled rod position correct classification rate was $99.9\%$. Compared to the misclassification frequencies reported in our previous work^13^, the results have improved, especially for the detection of empty grid positions in the VVER type of fuel (previously 35%, while now 88%). There is still room for improvement for VVER fuel, but the rates are much better than reported earlier. For BWR, the effect of burnable absorber rods cannot be excluded yet.

The misclassifications often occur in certain parts of the fuel grid, pointing towards structural characteristics. For GE-12 and GE-14 types of assemblies, most of the misclassifications occur in two separate grid locations, and for SVEA assemblies there are also two separate grid locations where the majority of the misclassifications happen. This would point towards a possible burnable absorber rod in these locations.

## Conclusion

The PGET method has previously been demonstrated to be able to detect partial diversion of nuclear material in a spent nuclear fuel assembly with very high precision^13^. In this work, we demonstrate a further improvement for fuel rod-level detection of anomalies in the central parts of the spent fuel. This improvement was achieved by using well chosen parts of the collected data, taking into account the specifics of the geometry of the imaged object in question. The see-through directions are shown to have much promise for contributing to high-quality images and will be further investigated with simulations and measurements with finer angular resolution. Data acquisition in different energy windows, namely in a 1200–1300 keV window capturing the Eu-154 isotope abundant in long-cooled spent nuclear fuel, is also proven to have promise for better quality data from the central parts of the fuel. Narrowing the energy window tightly around a gamma peak is shown to improve the results also for the conventionally used 662 keV gamma peak of Cs-137. The PGET method was demonstrated to be able to detect partial diversion of fuel material in the axial direction. Despite the relatively broad field of view of the device in the axial direction, partial absence of fuel rod material was detected as lower activity. Future measurements will be conducted with 0.5° angular resolution to investigate the see-through directions in more detail. The gamma energy windows used in data collection will be further narrowed down and more tests in the Eu-154 window will be done with a longer data acquisition time.

## Supplementary Information

Supplementary Information.

Supplementary Information.

Supplementary Information.

## Data Availability

The datasets used and/or analysed during the current study can be made available under a collaboration agreement with the Helsinki Institute of Physics and The Radiation and Nuclear Safety Authority of Finland. Please contact the corresponding author for more information.
