# Author Correction: Improved Passive Gamma Emission Tomography image quality in the central region of spent nuclear fuel

**DOI:** 10.1038/s41598-022-26119-9

**Published:** 2022-12-16

**Authors:** Riina Virta, Tatiana A. Bubba, Mikael Moring, Samuli Siltanen, Tapani Honkamaa, Peter Dendooven

**Affiliations:** 1 Helsinki Institute of Physics, University of Helsinki, Helsinki, Finland; 2 Radiation and Nuclear Safety Authority (STUK), Vantaa, Finland; 3 Department of Mathematical Sciences of the University of Bath, Bath, UK; 4 Department of Mathematics and Statistics of the University of Helsinki, Helsinki, Finland

Correction to: *Scientific Reports* https://doi.org/10.1038/s41598-022-16642-0, published online 21 July 2022

The original version of this Article contained an error in Reference 7, which was incorrectly given as:

Tobin, S. J. *et al.* Utility of including passive neutron albedo reactivity in an integrated NDA system for encapsulation safeguards. *ESARDA Bull.* **56**, 12–18 (2018).

The correct reference is listed below:

Tobin, S. J., Peura, P., Bélanger-Champagne, C., Moring, M., Dendooven, P., & Honkamaa, T. Measuring spent fuel assembly multiplication in borated water with a passive neutron albedo reactivity instrument. *Nuclear instruments & methods in physics research section a-Accelerators spectrometers detectors and associated equipment*. **897**, 32–37 (2018).

The original Article has been corrected.

