# Supplementary figures and images for: Improved Passive Gamma Emission Tomography image quality in the central region of spent nuclear fuel
Source: Sci Rep. 2022 Jul 21;12:12473. doi: 10.1038/s41598-022-16642-0 (PMC9304420; doi:10.1038/s41598-022-16642-0)

Supplementary Figure S1: Image  
quality index plot Loviisa 2020

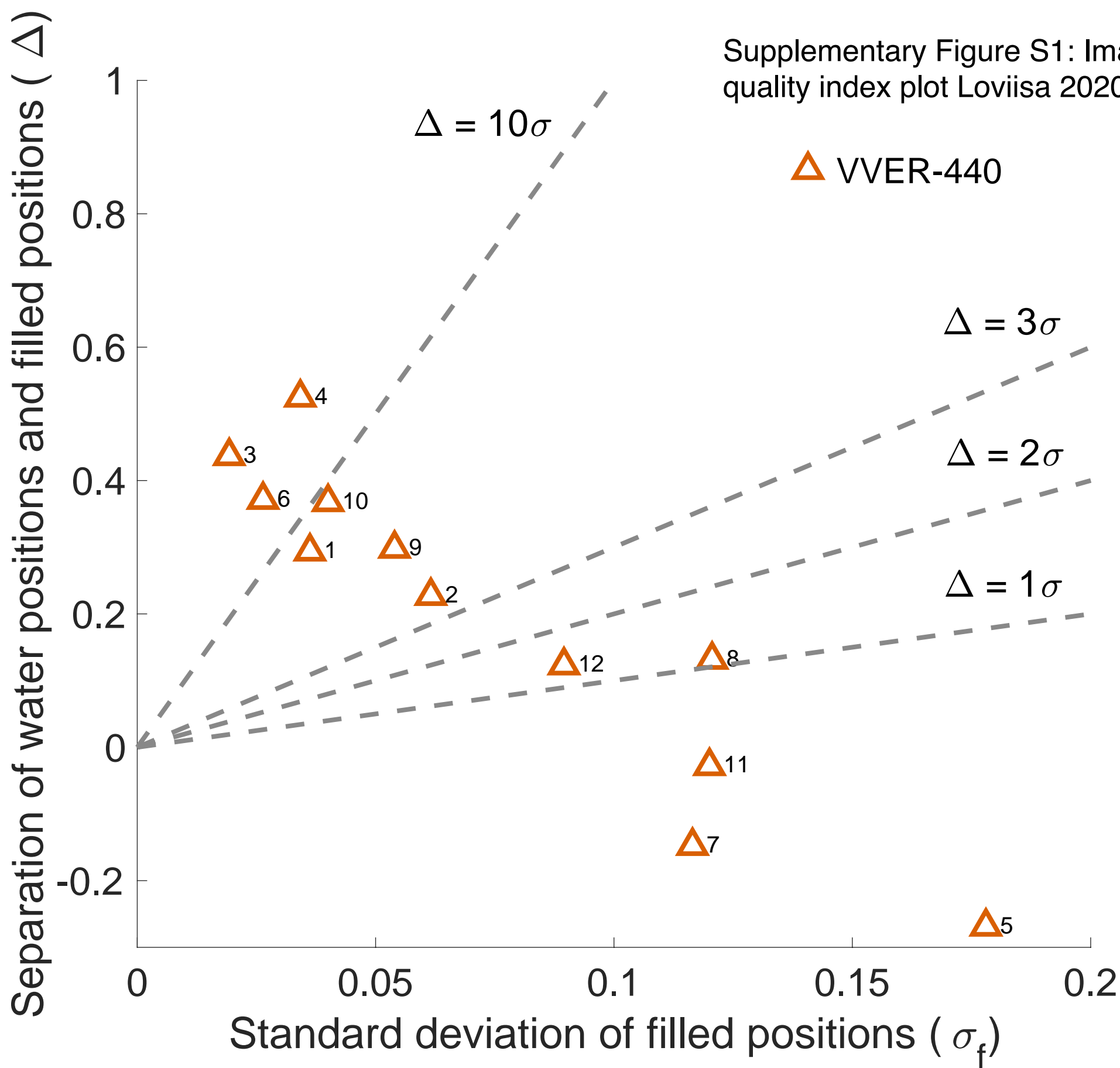

Supplement: Supplementary file 2 — Supplementary Information. [file 41598_2022_16642_MOESM2_ESM.pdf]

Supplementary Figure S2: Image quality index plot Olkiluoto 2021

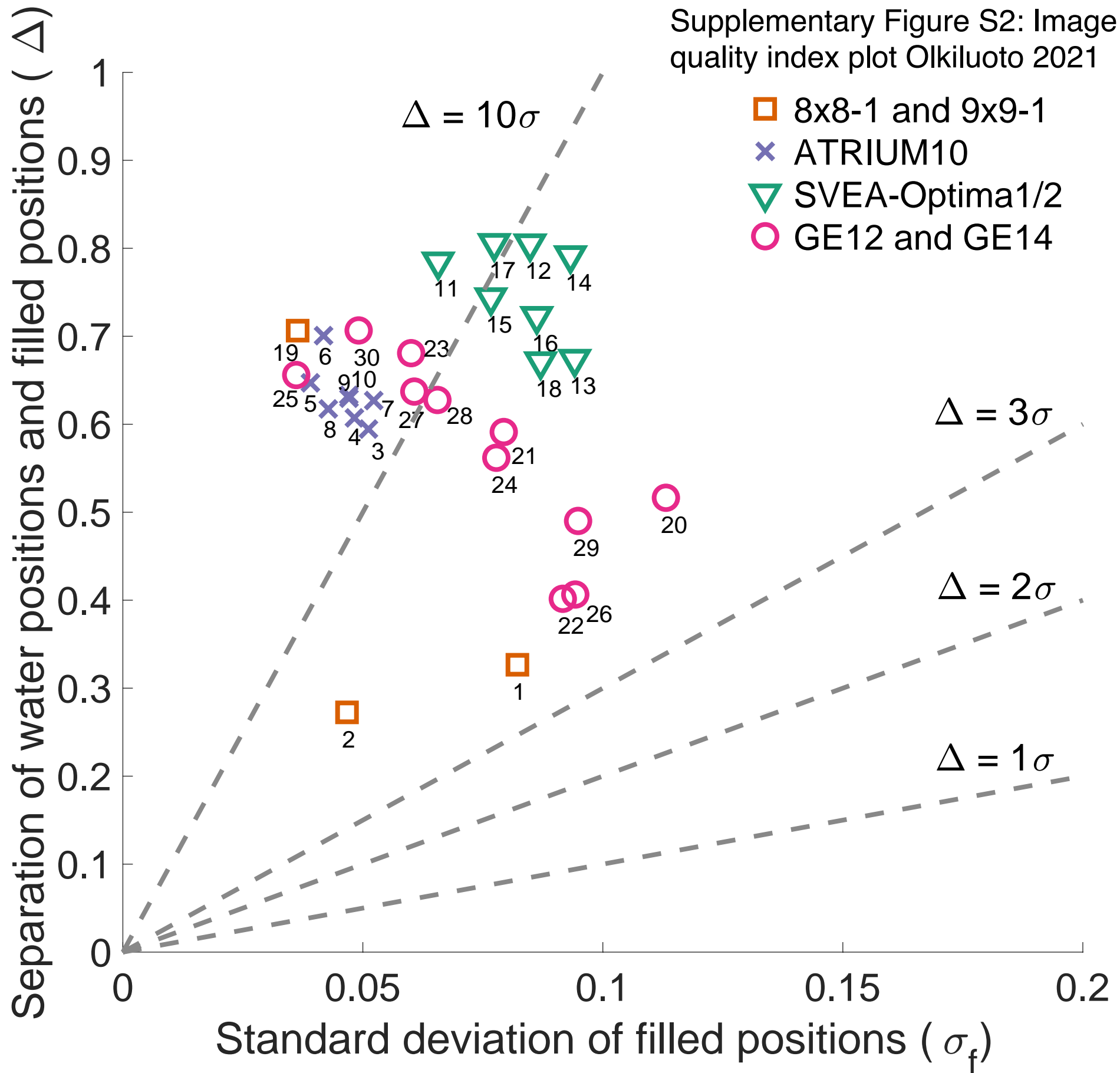

Supplement: Supplementary file 3 — Supplementary Information. [file 41598_2022_16642_MOESM3_ESM.pdf]
